# Expression of CRY2 Gene in the Brain Is Related to Human Navigation

**DOI:** 10.3389/fradi.2021.731070

**Published:** 2021-12-17

**Authors:** Shan Xu, Xiangzhen Kong, Jia Liu

**Affiliations:** ^1^Faculty of Psychology, Beijing Normal University, Beijing, China; ^2^Department of Psychology and Behavioral Sciences, Zhejiang University, Hangzhou, China; ^3^Department of Psychology and Tsinghua Laboratory of Brain and Intelligence, Tsinghua University, Beijing, China

**Keywords:** spatial navigation, CRY2, meta-analysis, Allen Human Brain Atlas, circadian rhythm

## Abstract

Navigation is a complex cognitive process. CRY2 gene has been proposed to play an important role in navigation behaviors in various non-human animal species. Utilizing a recently developed neuroimaging-transcriptomics approach, the present study reported a tentative link between the CRY2 gene and human navigation. Specifically, we showed a significant pattern similarity between CRY2 gene expression in the human brain and navigation-related neural activation in functional magnetic resonance imaging. To further illuminate the functionality of CRY2 in human navigation, we examined the correlation between CRY2 expression and various cognitive processes underlying navigation, and found high correlation of CRY2 expression with neural activity of multiple cognitive domains, particularly object and shape perception and spatial memory. Further analyses on the relation between the neural activity of human navigation and the expression maps of genes of two CRY2-related pathways, i.e., the magnetoreceptive and circadian-related functions, found a trend of correlation for the CLOCK gene, a core circadian regulator gene, suggesting that CRY2 may modulate human navigation through its role in circadian rhythm. This observation was further confirmed by a behavioral study where individuals with better circadian regularity in daily life showed better sense of direction. Taken together, our study presents the first neural evidence that links CRY2 with human navigation, possibly through the modulation of circadian rhythm.

## Introduction

Navigation is of pivotal importance for survival. Previous studies have suggested that spatial navigation is highly inheritable (1, 2) and genetic factors play a substantial role in susceptibility to navigation-related disorders such as Alzheimer's disease (3). Recent efforts have been made to identify the genetic basis of navigation ability in human (4, 5), and found that the expression map of S100 calcium-binding protein B (S100B) gene in the brain is specifically correlated with functional activation for scene perception, a core component of spatial navigation (4) and that functionally unstable APOE4 gene carriers lead to a boundary-driven error correction during wayfinding (5). Because navigation is a complex cognitive process that involves multiple cognitive functions, it is likely that more genes are involved in the process. Here, we investigate whether there are other genes to construct the gene repertoire specific for navigation along with the already known navigation-related genes such as APOE4, S100B, and TOMM40 (6).

One candidate is Cryptochromes that are highly conserved across animals and associated with navigation in insects and migratory birds [e.g., (7, 8)]. Although no direct evidence of Cryptochromes' involvement in human navigation has been reported, human Cryptochrome-2 (CRY2), one of the human Cryptochromes, can function as a replacement of Drosophila's inherent magnetosensor to restore the magnetic dependent navigation ability of genetically manipulated Drosophila (9), suggesting the functionality of CRY2 in navigation may be preserved in human as well. The underlying mechanism of CRY2 on navigation could involve, at least in part, two aspects. First, CRY2 might function as (a part of) the magnetosensor of a biological compass in human navigation as in non-human animals (10). In non-human animals, Cryptochromes are found to serve as a light-dependent magnetosensor [e.g., (7, 8, 11)], where the quantum spin dynamics of a radical-pair reaction in Cryptochromes change as a function of external magnetic fields and affect subsequent biochemical reactivity [e.g., (8, 11–13)]. Although magnetosensing in human remains controversial (14, 15), one study has reported that Cryptochromes, along with human ISCA1 gene, can form magnetosensing rod-like protein complex that exhibits spontaneous alignment in magnetic fields (10). Second, human Cryptochromes are known for participating in circadian regulation (16, 17), and human CRY2, along with CRY1, expresses rhythmically and binds with PER proteins to inhibit the positive limb of the feedback loop including CLOCK, ARNTL/BMAL1, and ARNT2/BMAL2 (16), which activates the transcription of core clock component genes and regulates the transcription of effector clock-controlled genes to further affect various physiological functions such as cellular metabolism (18, 19). In the present study, we first explored the relation between the CRY2 gene and human navigation, and then examined the potential involvement of the two functional aspects of the CRY2 gene in navigation.

To do this, we ran an integrative study by combining gene expression data in the human brain, large-scale neuroimaging meta-analysis activation maps, and behavioral test data to examine the potential link between CRY2 and human navigation. Specifically, we examined the pattern similarity of CRY2 expression in human brain and the brain functional activation in navigation-related tasks. A similar approach that combines neuroimaging and gene expression atlas [e.g., Allen Human Brain Atlas (AHBA)] has successfully linked genes with functional activation patterns (4), functional connectivity (20, 21), structural connectivity (22), and alterations in brain disorders such as schizophrenia (23) and autism spectrum disorder (24). To further illustrate the functionality of CRY2 in human navigation, we examined the correlation between CRY2 expression and various cognitive components underlying navigation, such as perception, memory, and action. Finally, we examined the relation between the neural activity pattern of human navigation and expression maps of genes of two CRY2-related pathways, i.e., the magnetoreception and circadian regulation.

## Materials and Methods

### Datasets

#### Allen Human Brain Atlas

Allen Human Brain Atlas (AHBA; http://www.brain-map.org) is a publicly available online resource for gene-expression data. The atlas characterizes gene expression [i.e., messenger RNA (mRNA) quantification] in postmortem human brain based on genome-wide microarray-based measurement, for more than 20,000 genes at ~500 sampling sites distributed over the whole brain [see (25) for more detail about the data collection]. Normalized expression data were used in the present study. To date (search conducted on March 30, 2017), six adult donors with no history of neuropsychiatric or neurological conditions were available in the database (ages 24, 31, 34, 49, 55, and 57 years; one female). Left hemisphere cerebral cortical data are available for all six donors, whereas right-hemisphere data are available for only two. Detailed information on donors and analysis methods is available at http://www.brain-map.org. Structural brain imaging data of each donor were used to align sampling sites into standard coordinate space.

#### Neurosynth

Neurosynth (http://neurosynth.org) is a platform for large-scale synthesis of task fMRI data (26). It uses text-mining techniques to detect frequently used terms as proxies for concepts of interest in the neuroimaging literature: Terms that occur at a high frequency in a given study are associated with all activation coordinates in that publication, allowing for automated term-based meta-analysis. Despite the automaticity and potentially high noise resulting from the large-scale meta-analysis, this approach has been shown to be robust and meaningful [e.g., (4, 21, 26, 27)], due to the high number of studies included.

#### Gene, Environment, Brain, and Behavior

Gene, Environment, Brain, and Behavior (GEB^∧^2) is a large-scale project aimed to investigate the associations among GEB^∧^2. The dataset used in the present study is part of the GEB^∧^2 project. The present study recruited 292 participants (155 females; mean age = 20.6 years, SD = 1.02 years) from Beijing Normal University (BNU), Beijing, China. Each participant completed the San Babara sense of direction questionnaire (SBSOD) and rated one's own circadian regularity in daily life.

We operationalized navigation ability as the sum scores of SBSOD. Previous studies have shown that people have explicit and accurate knowledge of their own navigation ability (28, 29). San Babara sense of direction questionnaire is a standard questionnaire on self-reported sense of direction in a large-scale environment, and has been widely used as a reliable proxy for navigation ability (30, 31). It includes 16 items, such as “I am very good at giving directions” and “I very easily get lost in a new city”. Participants were instructed to indicate the extent to which they agreed or disagreed with each statement on a five-point Likert-type scale. A higher total score indicates better performance in daily navigation, and thus better navigation ability.

The regularity of circadian rhythm was measured by a single question: comparing to people of similar age and educational background, do you keep regular hours in daily routine? The participants were asked to respond on a nine-point scale. The choices range from 1: very irregular to 9: very regular, a higher score indicating higher regularity.

All participants had no history of neurological or psychiatric disorders. The study was approved by the Institutional Review Board of BNU. Written informed consent was obtained from all participants before they took part in the experiments.

### Pattern Similarity Analysis of CRY2 Gene Expression in the Human Brain and Functional Activation Related to Navigation

We firstly generated a meta-analytic map for navigation using large-scale data from Neurosynth. The term “navigation” was used, which resulted in 61 studies with 2,989 activations in total. As expected, the meta-analytic activation map covers the well-established navigation-related regions, including the hippocampus, parahippocampal gyrus, and retrosplenial cortex [e.g., (4, 32–37)].

Next, we extracted CRY2 gene expression in the human brain from the AHBA. Three probes, A_23_P127394, A_23_P388027, A_24_P158587, were available for this gene in the atlas. Normalized CRY2 gene expression for each sampling site and each donor was obtained. Data of the three probes were averaged at each sampling site to estimate the expression level of CRY2. This provides a vector of the normalized gene expression values in a set of sampling sites, representing the spatial distribution of CRY2 gene expression across the brain for this donor.

To obtain the spatial correspondence between navigation-related activation and CRY2 gene expression, we first translated the location of each AHBA sampling site into MNI space with non-linear co-registration. We then performed an approximate random effect analysis implemented in alleninfo (38) to examine the pattern similarity of two maps. Briefly, we first correlated the spatial distribution of CRY2 gene expression of each donor with the statistic index of activation likelihood in navigation studies (i.e., the corresponding value in the navigation map), and reported a slope of best linear fit for each donor. We then performed a one-sample *t*-test on those estimates, to test whether the distribution of the slopes was significantly away from zero. Besides illustrating the correlation between CRY2 expression level and the probability of navigation-related activation across donors, the approximate random effect analysis allows estimating the generalizability of the observed correlation beyond the six donors.

Based on the random effect analysis, we evaluated the inter-donor repeatability of the CRY2-navigation correlation to determine whether the repeatability of a CRY2-activation correlation was statistically higher than that by chance. To do so we randomly sampled the same number of studies as in the above-mentioned navigation meta-analysis from the entire Neurosynth database, excluding those included in the navigation meta-analysis. We then generated the activation probability map for each sample and correlated this random-activation map with the expression map of CRY2, and counted how many donors' CRY2 expression level correlated significantly with the probability of the randomly pooled neural activation (uncorrected, two-tailed). This procedure was repeated 1,000 times, generating a null distribution of the inter-donor repetition. Without any link between CRY2 and navigation, the CRY2-navigation correlation should not deviate from the null distribution, i.e. with a threshold of α = 0.05, more than 5% of random samples should have equal or higher inter-donor repeatability than the correlation in question.

In addition, given the observed link between S100B gene and brain activity related to human navigation (4), we further tested whether CRY2 contributes to navigation independently or through its association with S100B. Specifically, we extracted the expression data of S100B from AHBA. Then, we conducted a stepwise multiple regression analysis with the probability of navigation-related fMRI activation at each sampling site as the dependent variable, and the respective expression levels of CRY2 and S100B at the same brain site, as well as their interaction term as independent variables. The interaction term was calculated as the product of the standardized expression levels of CRY2 and S100B. The regression was done separately for each donor of AHBA. We then examined the significance of coefficients of each independent variable across the donors using approximate random effect analysis. We further examined the statistical significance of each of the beta coefficients with the approximate random effect analysis across donors. When one regressor was not significant for one donor, we treated its beta value as zero.

### Association Between CRY2 Gene Expression With Neural Activity of Cognitive Processes Involved in Navigation

Navigation is a complex cognitive process, which involves multiple processes, for instance visual perception of key landmarks, memorizing the route, generating cognitive maps, selecting between alternative routes, etc. Next, we explored the underlying cognitive components that were modulated by the CRY2 gene.

To do so, we ran similar pattern similarity analysis between CRY2 gene expression and functional activation maps of candidate components. Here, we focused on activation maps of cognitive domains, such as perception, memory, and attention, rather than navigation itself. Note that this analysis was not intended to be a comprehensive survey, neither was it conducted in an exhaustive data-driven manner, and it was further limited by the availability of suitable key terms in Neurosynth database. Thus, in a half-heuristic-half-knowledge-driven manner, we chose nine broad domains which are supposedly involved in navigation and have been widely studied in psychology and cognitive neuroscience literature: perception (for instance, *object* and *sense perceptio*n during navigation), memory (e.g., the retrieval of *semantic* and *spatial memory* of the to-be navigated region), social cognition (e.g., the *self-referential* processes and *social interaction* involved in navigation), higher cognitive processes (such as *reasoning* and *decision making* in choosing alternative paths), language (e.g., *speech understanding* and *phonetic* processing required to understand instruction of a navigation task), attention (e.g., *spatial attention* and *attention shift* required to attending and orienting to important landmarks), executive functions (e.g., *response selection* and *inhibition* in an environment affording multiple action possibilities), motor (such as *eye movement* in the inspection of the environment and *motor control* in order to navigate through distance), and learning (such as *spatial learning* involved in navigation in novel environment). To further break down these broad domains, three sub-domains were selected from each domain (the italic terms mentioned above were some examples, see the Results section for a full list of subdomains), with a principle to cover both the spatial and non-spatial processes (for instance, *spatial memory* vs. *episodic memory* in Memory) in each domain whenever applicable, otherwise to cover both those more and less likely to be related to navigation (for instance, *scene perception* vs. *face perception* in Perception). Some sub-domains were identified by terms consisting of two words. Some of these two-word terms were built-in feature-terms in Neurosynth, and meta-analysis can be conducted based on these terms straightaway. For the two-word terms which were not built-in features in Neurosynth, studies were extracted by searching for those labeled by both one-word terms.

The activation map for each functional domain was generated using meta-analyses in the Neurosynth. The value in each voxel of the activation map of a given domain corresponds with the likelihood of this voxel being activated in studies recognized as relevant to that domain by Neurosynth. We ran the same pattern similarity analysis as the main analysis but used the activation map of each cognitive domain.

### Association of Navigation-Related Activation With Genes of CRY2-Related Pathways

Besides the exploration of cognitive components that might contribute to the CRY2-navigation link, we further investigated the functionality of CRY2 in navigation by examining the association of navigation with CRY2-related functions. Here, we focused on genes of the magnetoreception and circadian-regulation pathway (see section Introduction). A full list of examined genes is presented in Table 1. The pattern similarity between gene expression and neural activation was calculated with the same method for these genes as for CRY2. The robustness of these correlations was also evaluated by comparing the results with the null distribution. In doing so, we only considered a correlation robust when it yielded six repetitions across donors (among the top 5% in the null distribution), and those yielding five (among the top 7.4%) repetitions a marginal trend.

**Table 1:** Probe names of the genes tested in the present study.

**Category**	**Gene symbol**	**Gene name**	**Probe**
Gene of interest	CRY2	Cryptochrome circadian clock 2	A_23_P127394
			A_23_P388027
			A_24_P158587
Navigation	S100B	S100 Calcium binding protein B	A_23_P143526
			CUST_17042_PI4162
Iron-metabolism	ISCA1	Iron–sulfur cluster assembly 1 homolog, mitochondrial	A_23_P216766
			A_24_P387609
			A_24_P75543
			A_32, P25253
			CUST_7371_PI41626
Core clock components	CLOCK	Clock circadian regulator	A_23_P419038
			CUST_16994_PI4162
			CUST_190_PI416408
	PER1	Period circadian clock 1	A_23_P89589
			CUST_12126_PI4162
	PER2	Period circadian clock 2	A_23_P209320
			A_23_P411162
	PER3	Period circadian clock 3	A_23_P201461
			A_24_P230948
			A_24_P291231
	ARNTL	Aryl hydrocarbon receptor nuclear translocator-like	A_23_P162037 CUST_14423_PI4162
	CSNK1D	Casein kinase 1, delta	CUST_8506_PI4162
	CSNK1E	Casein kinase 1, epsilon	A_23_P29263
			A_23_P40664
			A_24_P918436
	ARNT2	Aryl-hydrocarbon receptor nuclear translocator 2	A_23_P83579 CUST_7028_PI41626
	NPAS2	Neuronal PAS domain protein 2	A_23_P218597
			A_23_P415984
	TIMELESS	Timeless circadian clock	A_23_P53276
			A_24_P231004

### Behavioral Correlation Between Navigation and Circadian Rhythm

Given the observed link between the circadian-related pathway and navigation-related brain activity, we hypothesized that circadian regularity in daily life could play a role in human navigation. To test this hypothesis, we collected measures of navigation ability and circadian regularity in a large sample of the GEB^∧^2 project, and ran a partial correlation analysis of these two measures. Participant's sex was controlled for in the analysis.

## Results

**Figure 1:**
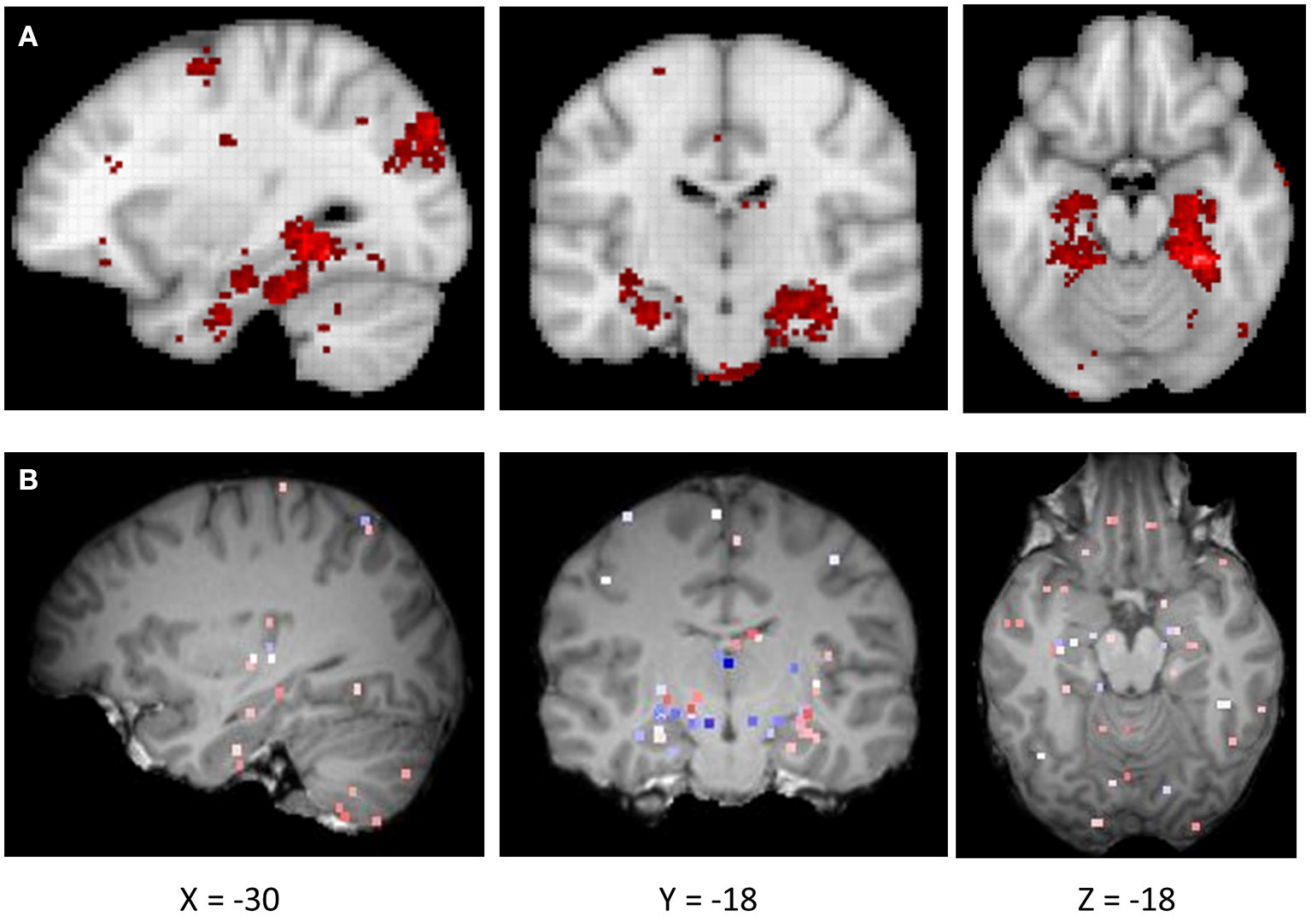
**(A)** The activation probability (association test) maps for navigation derived from the Neurosynth-based meta-analysis. **(B)** Gene expression map of CRY2 of a representative donor (donor ID: H0351.2002) from AHBA.

**Figure 2:**
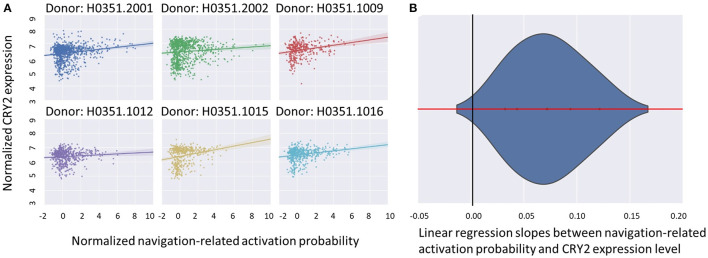
The correlation between CRY2 expression level and the probability of navigation-related neural activities. **(A)** The scatter plots illustrating the correlation of each donor. The X axis denotes the standardized probability of navigation-related activity, while the Y axis denotes the standardized CRY2 expression level. Each point in the plot denotes a sample site in AHBA. The positive correlation illustrated in **(A)** suggested that the expression level of CRY2 is positively correlated with the probability of navigation-related activation across the brain. **(B)** The approximate random effect analysis estimated the distribution of the linear regression slopes between navigation-related activation probability and CRY2's local expression level, the X axis being the value of the slope, and the Y axis the probability density of a given slope value. The zero of the X axis (denoted by the black vertical line) falls into the extreme left end of the distribution, suggesting the significance of the examined random effect.

We first examined the similarity between the expression map of CRY2 in the brain and the map of navigation-related activation (Figure 1; see section Materials and Methods). Results showed a positive correlation between these two maps, which was reproducible across all six donors of AHBA (Figure 2; *Mean*_beta values_ = 0.07, *SD* = 0.03; *Mean*_*r*_ = 0.17, *SD* = 0.06; *p*s < 0.045, *SD* = 0.018). Moreover, the approximate random effect analysis confirmed this correlation [Figure 2B, *t*_(5)_ = 5.46, *p* = 0.003], suggesting that the significant correlation could be generalized beyond the six donors. To examine the robustness of the observed correlation, we established a null distribution of inter-donor repetition of gene expression-navigation correlation, that is, the number of donors whose CRY2 expression level significantly (uncorrected, two-tailed) correlated with the activation map of a random sample of fMRI studies. The null distribution was positively skewed (*M* = 1.4, *SD* = 1.7). Importantly, we found that the number of repetitions among donors (six out of six) in the CRY2-navigation correlation were among the extreme 3.1% in this distribution, which suggested that this correlation was unlikely to be caused by chance (*p* = 0.031). In short, regions with a higher level of CRY2 gene expression were more likely to be activated in navigation-related tasks, suggesting a tentative link between the CRY2 gene and human navigation.

We also found that the expression level of CRY2 across the brain was negatively correlated with that of the navigation-related S100B for each donor [beta_average_ = −0.34, *t*_(5)_ = 7.31, *p* = 0.001, Supplementary Figure 1]. To rule out the possibility that CRY2 contributed to navigation through its association with S100B, we conducted a step-wise regression analysis (see section Materials and Methods), and found that, for all the donors, the resultant models were significant ($Mean\;R_{\text{adjusted}}^{2}$ = 0.059, *SD* = 0.025, *p*s < 0.02). Specifically, the standardized expression level of CRY2 (*Mean*_standardized beta_ = 0.11, *SD* = 0.10) entered the resultant models for four out of six donors (*p*s < 0.045), the standardized expression level of S100B (*Mean*_standardized beta_ = −0.16, *SD* = 0.09) was accepted for five out of six donors (*p*s < 0.002), while the interaction term was significant for two out of six donors (*p*s < 0.009). Pulling together the beta coefficients of all donors, the approximate random effect analysis revealed that the beta coefficient of expression levels of CRY2 was significantly larger than 0 [*t*_(5)_ = 2.87, *p* = 0.035] and those of S100B smaller than 0 [*t*_(5)_ = −4.30, *p* = 0.008]. In contrast, the beta coefficients of CRY2-S100B interaction term was not significantly different from 0 (*p* = 0.175). These results together suggested that CRY2 and S100B independently contributed to navigation-related activation.

**Figure 3:**
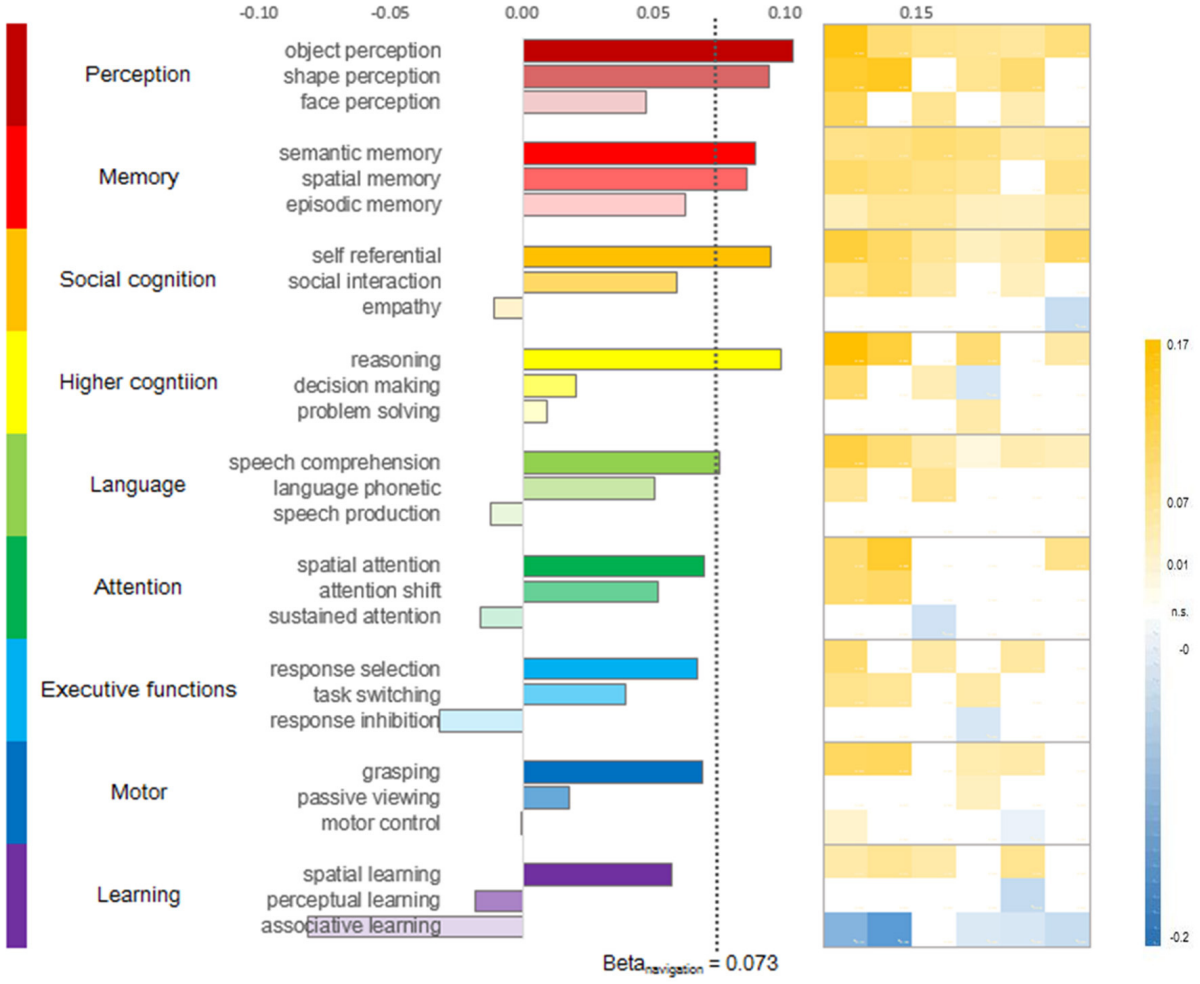
CRY2 expression-activation correlation in various cognitive domains. Sub-domains of perception and memory strongly correlated with CRY2. In contrast, the domain of higher-level cognition and social cognition displayed variation in terms of CRY2-activation correlation. The domains of Learning, attention, reasoning and response control did not show similar correlation with CRY2. **(Left)** Averaged beta for the correlation between CRY2 expression level and the activation probability of respective function domain (three representative sub-domains presented). The dashed line denotes the beta value of the CRY2-navigation correlation. **(Right)** The beta matrix of CRY2-activation correlation across donors. Each column shows the beta values of one donor, and each row shows the beta values of the correlations between each donor's CRY2 expression level and the activation probability of the function domain denoted by the corresponding bar on the left across the brain.

To further explore which component of navigation is associated with CRY2, we examined nine domains (i.e., perception, memory, social cognition, intelligence, language, attention, executive functions, motor, and learning) that may be relevant to navigation. Specifically, we examined the correlation between CRY2's expression map with the fMRI activation map of each of these domains extracted from the Neurosynth database (see section Materials and Methods). The results were presented in Figure 3. In general, we found that the expression level of CRY2 correlated with neural activity in multiple cognitive domains. For instance, correlations in the Memory domain generally yielded high and robust correlations, while the correlation in the domains of Perception and Social cognition was restricted to certain sub-domains (e.g., object and shape perception in perception, and self-reference in social cognition). In contrast, in the domains of Motor and Executive function, the correlations were generally low. In addition, there was within-domain variation. For instance, in the domain of Intelligence, reasoning yielded high and robust correlation, while decision-making and problem-solving were not robustly correlated with CRY2. In short, instead of being associated with a specific aspect of navigation, CRY2 seemed to contribute to navigation through multiple pathways. This possibility is particularly interesting since previous studies have shown that CRY2 is involved in multiple functions, such as magnetoreception and circadian-related functions. Next, we examined the relation between navigation and different functions of CRY2.

**Figure 4:**
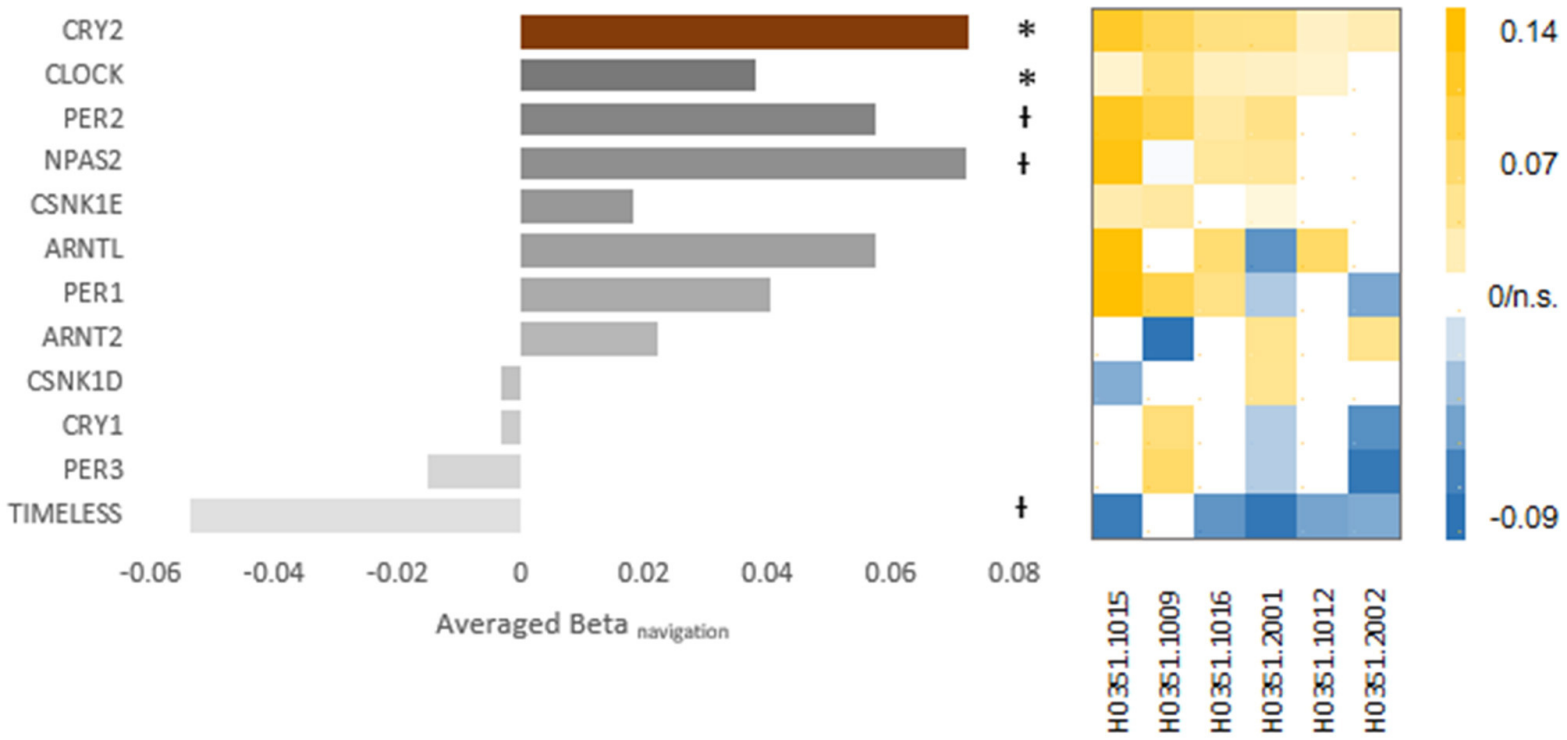
The expression-navigation correlation of other core-clock component genes. The bar chart shows the averaged beta of the expression-navigation correlation of each core clock component gene. The brown bar on top denotes the correlation of CRY2. The asterisks (*p* < 0.05) and the crosses (*p* < 0.10) denote the uncorrected *p*-values of random effect analysis. The beta-value matrix (right) illustrates the robustness of the respective correlation. Each column shows the beta values of one donor, and each row represents the same gene represented by the corresponding bar on the left. The color of each block represented the value of the respective beta, while blocks corresponding to insignificant correlations were left blank.

As for the circadian aspect of CRY2, we focused on a set of circadian regulation genes (i.e., core clock component genes), including CLOCK, CRY1, ARNTL, PER1, PER2, PER3, CSNK1D, CSNK1E, NPAS2, TIMELESS, and ARNT2 (for details, see Table 1 for the list of full names of genes and probes used in examining the expression levels). Four genes showed significant correlation (Figure 4): CLOCK, averaged beta = 0.038, *p* = 0.006, repeated in five out of six donors; PER2, averaged beta = 0.057, *p* = 0.045, repeated in four out of six donors; NPAS2: averaged beta = 0.072, *p* = 0.013, repeated in four out of six donors; TIMELESS: averaged beta = −0.054, *p* = 0.030, repeated in five out of six donors. The navigation-expression correlation was not significant for the rest core clock component genes (CRY1, ARNTL, PER1, PER3, CSNK1D, CSNK1E, and ARNT2) (*p*_*uncorrected*_s > 0.10). Among the genes that showed significant correlation, the CLOCK gene survived FDR correction for the multiple comparison correction (*q* = 0.05).

**Figure 5:**
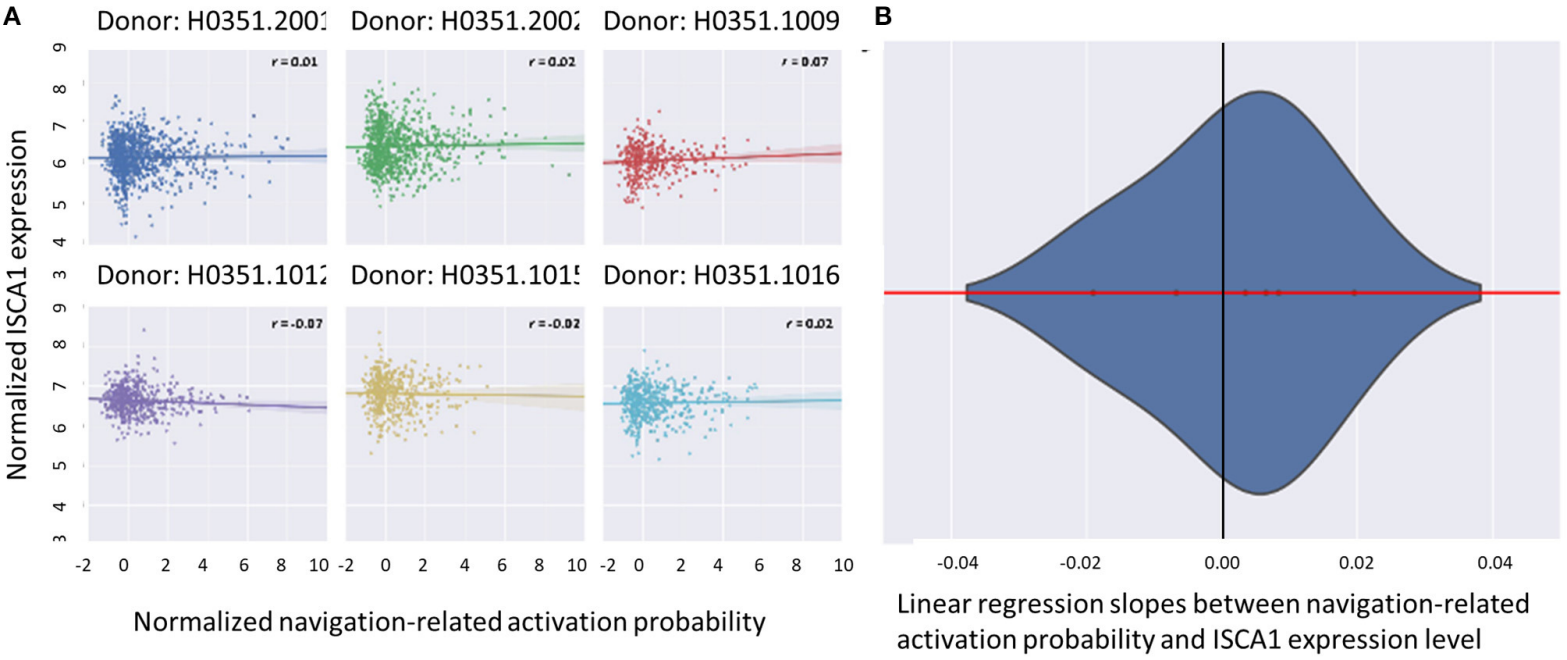
**(A)** The correlation between the probability map of navigation-related neural activity and the local expression level of ISCA1 was not apparent for any of the donors. The X axis denotes the standardized probability of navigation-related activity, while the Y axis denotes the ISCA1 expression level. Each point in the plot denotes a sample site in AHBA. **(B)** This correlation was not significant across donors in the approximate random effect analysis. The Y axis denotes the probability density of the estimated linear regression slopes between standardized navigation-related activation probability and ISCA1 local expression level, while the X axis denotes the value of the slope. The zero of the X axis did not fall into the extreme 5% of the distribution, suggesting the insignificance of the examined random effect.

As for the magnetoreception aspect of CRY2, we focused on ISCA1 gene that has been proposed to form magnetoreceptor complex with CRY2. The expression map of this gene showed no significant correlation with the activation map of navigation (Figure 5).

**Figure 6:**
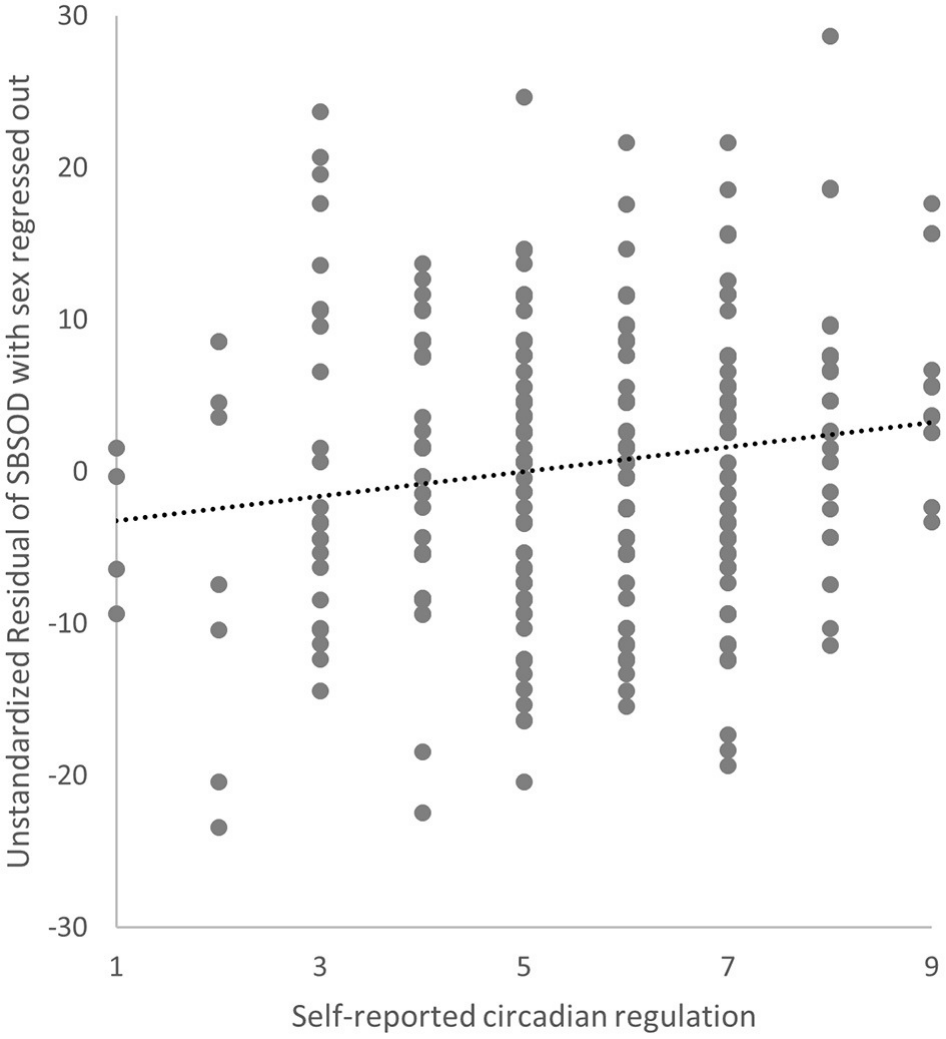
A positive correlation between self-reported circadian regularity and navigation ability measured by SBSOD with sex regressed out. Each dot represents one individual. X axis denotes the score of self-reported circadian regulation, and Y axis denotes the score of sense of direction after regressing out sex.

In short, it seems that the circadian aspect, not the magnetoreception aspect, of CRY2 was related to human navigation ability. Based on this observation, we predicted that individuals with better circadian regularity likely possess better navigation ability. Indeed, college students' self-reported circadian regularity was positively correlated with self-reported sense of direction (indexed by the SBSOD score), a core component of navigation, after controlling for sex (*r* = 0.15, *p* = 0.009; Figure 6).

## Discussion

In this study, we established a tentative link between the CRY2 gene and human navigation. First, we found a significant similarity between the CRY2 expression map and fMRI activation map of navigation in the human brain. Further analyses revealed that the CRY2 gene modulated navigation possibly through various cognitive components of navigation, such as object perception and spatial memory. Moreover, instead of the speculated magnetoreception aspect, the link between CRY2 and navigation was likely based on the circadian-related function of CRY2, as the pattern similarity between human navigation and the CLOCK gene, a core circadian regulator gene, was observed. A follow-up behavioral study confirmed this finding by showing that individuals with better circadian regularity tended to have better sense of direction in daily life. In sum, our study provides the first empirical evidence linking the CRY2 gene to human navigation.

The present study demonstrated that CRY2's expression was spatially correspondent to navigation-related neural activity. However, since navigation is a complex cognitive process and involves various cognitive components, we then investigated through which of these cognitive components CRY2 affects navigation. To do this, we conducted an exploratory analysis to examine the relation between CRY2 expression and several major cognitive domains presumably related to human navigation to varying extents. We found that CRY2 correlated with multiple cognitive domains, suggesting that CRY2 might be functionally related to multiple components of navigation. Further, CRY2's expression level correlated with functional activation in different domains with different degrees of robustness as well as within-domain variation, and such variation is consistent with the presumable involvement of specific domain or subdomains in typical navigation tasks. For instance, in the memory domain CRY2 yielded robust correlations in multiple sub-domains (e.g., spatial memory and semantic memory), presumably due to the close functional relevance between navigation and memory. In contrast, the correlations in perception and social cognition were restricted to subdomains presumably relevant to navigation. For instance, in the domain of Perception, object perception that is related to scene and landmark perception in navigation yielded robust correlation; in contrast, face perception was not robustly correlated with CRY2 expression. In addition, self-referential in the social cognition domain had robust correlation with CRY2, probably due to the involvement of egocentric navigation (39–41); however, the same correlation did not exist for empathy, which is also theoretically less relevant to navigation. Taken together, the analysis on related cognitive domains suggested CRY2's association with navigation is functionally specific. Note that CRY2 has been found peak regulated in the perinatal brain (42). This suggests that the cortical development during perinatal period might be critical for navigation-related behaviors which only become observable in later developmental stages. Future studies might benefit from integrating the developmental perspective with analyses in the spatial domain such as those in the present study.

Our study further demonstrated that the association between the CRY2 gene and navigation might be realized through circadian-regulation, not magnetosensing-related, function of CRY2. Previous studies have shown that circadian regulation is a well-established function of human Cryptochromes (16, 17), and human CRY2 is a negative regulator in the transcription-translation negative feedback loop in the molecular clock (43–45), which presents widely in mammalian tissues (45–47). Indeed, CRY2 affects various physiological functions regulated by the circadian system [e.g., (48–50)]. In our study, we found that four out of eleven of the core clock component genes, especially the CLOCK gene, showed the trend of correlation with navigation-related neural activities in the brain, suggesting a link between navigation-related neural activities and the transcriptional-translational feedback loop of circadian regulation. This link was further confirmed by the behavioral correlation between navigation ability and circadian regulation. However, note that CRY2 showed higher correlation with navigation-related neural activity than CLOCK. We speculate that this difference is the result of the functional specificity of CRY2 among circadian regulation genes. For instance, the regulation of glucose homeostasis and immune function has been found relatively specific to Cryptochromes, but not apparent for other clock genes, and the relatively high expression level of CRY2 in brain regions involved in navigation might be a result of the high demand of metabolic regulation in these cortical regions, given the substantial cognitive demands in complex navigation tasks. In other words, instead of navigation ability being a readout of the entire transcriptional-translational feedback loop of circadian regulation, CRY2 might affect certain biochemical pathway in neurons involved in navigation via its rhythmic expression and its unique biochemical properties, and the CLOCK-navigation correlation might be a result of the direct transcriptional modulation between CLOCK and CRY2 (43, 44, 47).

In contrast to circadian regulation, we did not find evidence for the CRY2 gene affecting human navigation via magnetosensing. For non-human animals, Cryptochrome's role as a light-dependent magnetosensor has been well-investigated [e.g., (7, 8, 11)] and influential models were proposed based on the quantum spin dynamics of a radical-pair reaction in Cryptochromes change in response to external magnetic field [e.g., (8, 11–13)]. However, magnetosensing in human has been controversial (14, 15). Intriguing results have been reported suggesting that human CRY2, despite having not been demonstrated *in vivo*, possesses the biochemical properties necessary for sensing magnetic field, and might still have the functional potential for this purpose (9, 10). Our study, however, did not support the conjecture that CRY2 affects navigation via magnetosensing, as we failed to find the link between the ISCA1 gene, which proposedly forms the magnetosensing complex with CRY2 as potential human biocompass (10), and navigation-related neural activity in the human brain. Note that the present study only examined the expression of the ISCA gene in the brain because of the availability of the data, and future studies with more comprehensive data are needed to examine the relation between the magnetosensing function of CRY2 and navigation in other tissues, such as retina, where the photo-dependent cryptochrome-involved magnetoreception is likely to take place.

Combining gene expression data in the human brain, large-scale neuroimaging meta-analysis activation maps, and behavioral test data, the present study showcased how a cognitive imaging genetics approach can be used to explore the genetic basis of cognitive functions. However, several limitations shall be noted, which shall be addressed in future studies. First, our study is mainly based on correlational analyses, and the exact biochemical pathway underlying such correlation is unclear, and more genetic evidence is needed to support the present interpretations. Therefore, the cognitive imaging genetics approach used in this study can only serve as an exploratory tool, which provides clues for future fine-tuned studies. Second, in this study we relied on a meta-analysis approach to obtain brain activation map for navigation as well as for other cognitive domains. This approach, on one hand, provides a quick-and-dirty way of extensively exploring multiple cognitive functions with reasonable reliability; the results from meta-analysis, on the other hand, is unlikely to fully capture the fine structure of cognitive functions because the representativeness of available literature and the deviation between conceptual and operational definitions of cognitive functions is in question. Therefore, the finding from our study is tentative, which provides testable hypotheses for more focused and more hypothesis-driven studies.

## Data Availability Statement

The data analyzed in this study is subject to the following licenses/restrictions: The GEB^∧^2 dataset used in this study are available on request to the corresponding author. Requests to access these datasets should be directed to Jia Liu, liujiathu@tsinghua.edu.cn.

## Ethics Statement

The studies involving human participants were reviewed and approved by the Institutional Review Board of Beijing Normal University. The patients/participants provided their written informed consent to participate in this study.

## Author Contributions

SX and JL contributed to conception and design of the study. SX performed the statistical analysis and wrote the first draft of the manuscript. All authors contributed to manuscript revision, read, and approved the submitted version.

## Funding

This study was funded by the National Natural Science Foundation of China (31861143039 and 31600925), the Fundamental Research Funds for the Central Universities (2019NTSS27 and 2021XZZX006), Shenzhen Institute of Artificial Intelligence and Robotics for Society (AC01202005022), and the National Basic Research Program of China (2018YFC0810602).

## Conflict of Interest

The authors declare that the research was conducted in the absence of any commercial or financial relationships that could be construed as a potential conflict of interest.

## Publisher's Note

All claims expressed in this article are solely those of the authors and do not necessarily represent those of their affiliated organizations, or those of the publisher, the editors and the reviewers. Any product that may be evaluated in this article, or claim that may be made by its manufacturer, is not guaranteed or endorsed by the publisher.
