# Supplementary material for: Expression of CRY2 Gene in the Brain Is Related to Human Navigation
Source: Front Radiol. 2021 Dec 17;1:731070. doi: 10.3389/fradi.2021.731070 (PMC10365100; doi:10.3389/fradi.2021.731070)
Supplement: Supplementary file 1 [file Data_Sheet_1.docx]

Supplementary Material

# Supplementary Figures


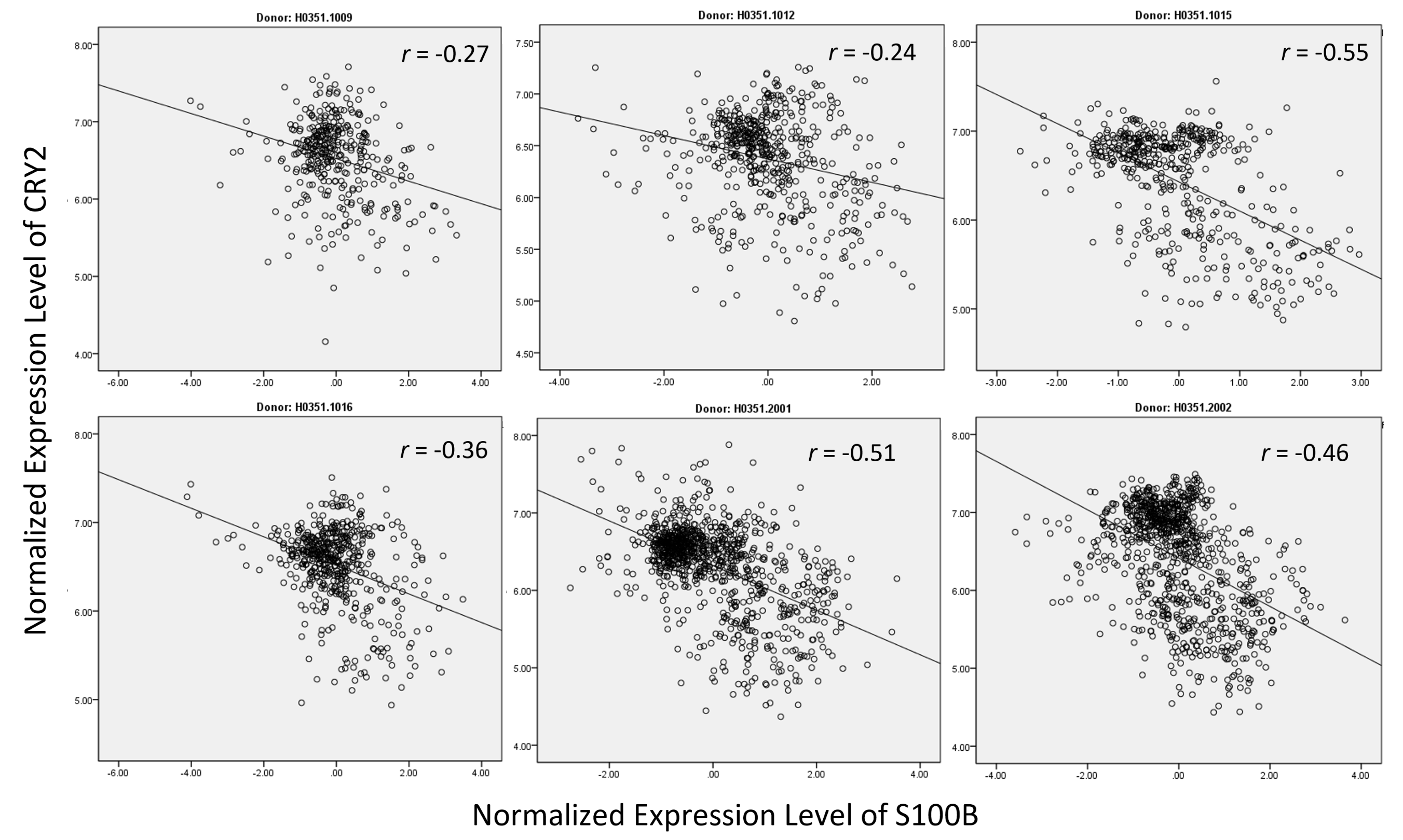


**Supplementary Figure 1. Correlation between the Normalized expression level of S100B and CRY2 for each donor.** The X and the Y axis denotes the normalized expression level of S100B and CRY2 respectively, and each point in the plots represents the data from a sampling site of that donor.
